# Stigma, mental illness & ethnicity: Time to centre racism and structural stigma

**DOI:** 10.1111/1467-9566.13615

**Published:** 2023-02-03

**Authors:** Dharmi Kapadia

**Affiliations:** ^1^ Department of Sociology The University of Manchester Manchester UK

**Keywords:** ethnicity, mental health services, mental illness, racism, stigma, structural stigma

## Abstract

This article critically reviews previous research in the field of stigma, mental illness and ‘race’ and ethnicity, and argues for a shift of focus from individual and community blame, as inferred by mental illness stigma, to a more comprehensive view of how stigma operates against a backdrop of structural and institutional racism. Ethnic minority people have poorer mental health outcomes compared with White majority populations. Dominant narratives of greater mental illness stigma in ethnic minority populations, due to religious, spiritual or traditional beliefs and leading to a lower use of services, have taken centre stage in the explanations for these consequent poorer outcomes. This article argues that this ‘fact’ has become taken for granted as knowledge without corresponding comparative research evidence. Research in the field has also failed to robustly consider how racism might operate in conjunction with different forms of mental illness stigma (particularly structural stigma) to exacerbate mental illness and influence pathways to mental health treatment. Future research should centre the role of racism and structural stigma in explaining the poorer mental health outcomes for ethnic minority people.

## INTRODUCTION

In recent years, particular narratives and discourses around the greater mental illness stigma experienced by ethnic minority people have emerged. Simply put, the narratives assert that due to certain religious, spiritual and cultural beliefs held by ethnic minority populations, there are greater levels of mental illness stigma in these populations that deter help‐seeking, and ultimately lead to more severe mental illness and poorer mental health outcomes. These narratives can be seen in the work and accounts of psychiatrists, academics, voluntary organisations and people with lived experience. However, there is little evidence that ethnic minority people’s poor mental health outcomes are mainly due to the apparent greater stigma felt by ethnic minority people. We have also seen how structural, institutional and interpersonal racism (Nazroo et al., 2020) have negatively affected both ethnic minority people’s mental health (Wallace et al., 2016) and their treatment outcomes (Kapadia et al., 2022). However, the way in which racism may shape stigma narratives about ethnic minority people has not been considered.

This article critically reviews the previous research in the field of stigma, mental illness and ethnicity, drawing predominantly on research that has been conducted in the UK and the USA, reflecting the geographical concentration of where research has taken place. It goes on to discuss the way in which narratives about stigma in ethnic minority populations have perpetuated the racialisation of ethnic minority groups. Finally, this article sets out recommendations for the future of research on ethnic inequalities in mental illness stigma, arguing for (1) a need to appropriately situate the role of stigma within the broader context of racism and racial inequalities in mental illness and the use of mental health services, (2) well‐designed comparative (with White majority populations) qualitative work that allows us to truly evaluate the difference in the nature and effect of mental illness stigma between ethnic groups, and appropriately designed quantitative empirical approaches to allow us to assess the magnitude of the postulated greater mental illness stigma within ethnic minority populations and (3) an examination of where stigma narratives have originated from and for what purpose, in order to make an attempt at disrupting them.

## THE DAMAGING NATURE OF STIGMA

The stigma associated with having a mental illness is a major burden affecting people across the globe. The World Psychiatric Association claims that mental illness stigma is the ‘single most important barrier to quality of life of mental health consumers and family members—more so than the illness itself—and a major impediment to mental health reform and development’ (World Psychiatric Association, 2019). The damaging nature of stigma was further consolidated in the recent Lancet Commission on Ending Stigma and Discrimination in Mental Health (Thornicroft et al., 2022), which stated that mental illness stigma ‘contravene[s] basic human rights and [has] severe, toxic effects on people with mental health conditions that exacerbate marginalisation and social exclusion’ (p. 1438). Accordingly, The World Health Organisation has stated in its Mental Health Action Plan, a vision for a ‘world in which mental health is valued, promoted and protected…[ ]… in order to attain the highest level of health and participate fully in society and work, free from stigmatisation and discrimination’ (World Health Organisation, 2021, p. 4).

Over the past 20 years in the UK, there have been large‐scale attempts by The Department of Health, The Royal College of Psychiatrists and charities to address the stigma that people with mental illness face from members of the public, family and friends and institutions. The most notable anti‐stigma campaigns, Changing Minds (Crisp et al., 2004), Time to Change (Henderson & Thornicroft, 2009) and Heads Together (Heads Together, 2018), have had some success in opening up conversations about mental health and illness and reducing public stigma (Henderson et al., 2016). Unsurprisingly, mental illness stigma continues to attract and warrant attention from government, the public, patients and health researchers, due to the undeniable research evidence that shows that people facing mental illness stigma have worse life chances than those people not affected by mental illness (Corrigan, 2004; Corrigan et al., 2014; Phelan et al., 1998; Schomerus & Angermeyer, 2008; Thornicroft, 2006, 2008). Moreover, the life disadvantages that are seen because of mental illness stigma are over and above the reduced life chances that poor mental health alone confers (Link & Phelan, 2001).

Sociological theorisations about mental illness stigma, both its meaning and effects, are well developed in the field. Goffman’s groundbreaking work on stigma has inspired 6 decades of work in the sociology of mental health (as well as in the sociology of stigma). Defining stigma as ‘an attribute that is deeply discrediting’ (Goffman, 1963, p. 13) that serves to ‘disqualif[y]’ a person or a group of people ‘from full social acceptance’ (Goffman, 1963, p. 11), Goffman considers attitudes such as these to be deeply harmful to those that are stigmatised, stating, ‘by definition, of course, we believe the person with a stigma is not quite human. On this assumption, we exercise varieties of discrimination, through which we effectively, if often un‐thinkingly, reduce his life chances’ (1963, p. 15). Goffman’s work on stigma is, for the most part, individualistic in its approach to how stigma operates in social interactions (Tyler, 2020); that is, stigma operates in relationships between people and this is how it is produced, maintained and reproduced. There is much less consideration of how social structures help to produce and strengthen stigmatising attitudes and how those in powerful social roles are able to stigmatise others to a greater extent than those without power. The ‘astructural’ (Bruckert & Hannem, 2012, p. 1) nature of Goffman’s work has been addressed by many scholars in relation to mental illness stigma. For example, Link and colleagues’ modified labelling theory (Link et al., 1989) and Pescosolido’s network theory on stigma (Pescosolido & Martin, 2015) are examples of more comprehensive theories of how mental illness stigma works as a mechanism in personal interactions, across social boundaries and in reproducing power relationships. Most recently, Tyler and Slater (2018) have reconceptualised stigma by emphasising the political and cultural power that is yielded by those that deploy it for their purposes, and have brought to the forefront issues surrounding how stigma is used as social control.

Where ethnic minority groups are concerned, there is some evidence that there is greater mental illness stigma compared with White majority groups, but in much of this evidence (e.g., Bhavsar et al., 2019; Eylem et al., 2020), the role of stigma is not appropriately considered alongside structural determinants of poor mental health outcomes. Specifically, the institutional racism evident in the psychiatric and psychological professions (Fernando, 2017; Kapadia et al., 2022; Nazroo et al., 2020) has not been linked in any depth to the literature on stigma to consider the similarities between the two systems of power (stigmatisation and racism) and how both impact negatively on ethnic minority people. In addition, the conversations around stigma brand ethnic minority people not only as ‘the stigmatised’ but also as ‘the stigmatisers’. This is in line with how all people with mental illness are branded: both as the victims of stigma and situated as part of the general public that holds stigmatising attitudes (Pescosolido & Martin, 2015). This ignores the fact that social structures produce and strengthen stigmatising attitudes and those in powerful social roles are able to stigmatise others to a greater extent than those without power (Link & Phelan, 2014; Tyler, 2020). Previous historical and contemporary work has highlighted the often intentional and harmful stigmatisation both initiated and propagated by the psychiatric profession (partly via anti‐stigma campaigns) in order to raise the professional status of this medical speciality (Long, 2014; Pilgrim & Rogers, 2005; Schulze, 2007), although the focus of these studies has not been on ethnic minority groups.

## ETHNICITY, RACE, RACISM AND RACIALISATION—WHAT DO THEY MEAN?

Before delving into the main arguments of the article, I outline brief definitions of the way I am using ethnicity, ‘race’, racism and racialisation throughout. Ethnicity refers to a number of aspects of a person’s identity including, but not limited to, country of birth, parents’ country of birth, languages spoken, cultural and family practices, skin colour, dress, religion and diet. Additionally, there is an assumption of common origins (Bradby, 1995) for people belonging to the same ethnic group, but in reality people belonging to the same socially constructed ethnic group can have very different migration and family histories; for example, some people classified as ‘South Asian’ in the UK may have migrated from East Africa. Throughout the article, I use ethnic minority group, people or populations to refer to people who are subject to marginalisation based on their ethnic background. In some countries, instead of the term ‘ethnicity’, ‘race’ is used instead. Generally, when used in official or administrative data (e.g., in the United States of America), ‘race’ is used as a synonym for ethnic group and does not refer to the historical notion of biological race (i.e., the idea that there are real genetic differences between, for example, Black people and White people [postulated as two different ‘races’]), which has been widely discredited. Accordingly, ‘race’ is always written in scare quotes to signify that it refers to socially constructed categories of ‘race’ not real biological races (Delgado & Stefancic, 2017).

Racism is a widespread system of unfairly advantaging some ethnic groups whilst disadvantaging other ethnic groups based on their ethnic or racial background (Bonilla‐Silva, 1997). Racism can be structural, institutional or interpersonal in nature (Jones, 2000; Nazroo et al., 2020). Structural racism refers to the processes that lead to disadvantage in accessing economic, physical and social resources; institutional racism is legitimised by discriminatory policies and norms embedded in large institutions (such as the National Health Service) and captures a broad range of practices that perpetuate differential access to services and opportunities within institutions; interpersonal racism refers to discriminatory treatment during personal interactions, such as verbal or physical abuse, but also refers to acts of ignoring or avoiding people due to their ethnic background. Finally, racialisation (within the field of medical sociology) refers to the problematic assumption of ascribing ethnic minority people’s health status, health behaviours and health outcomes to be caused by simply *belonging to a certain ethnic group* (Kapadia & Bradby, 2021; Nazroo, 1998). There are examples of how ethnic minority people have been racialised in the process of diagnosis and treatment, later in this article.

## MENTAL ILLNESS STIGMA IN ETHNIC MINORITY GROUPS—WHAT DO WE KNOW?

The poor mental health of many ethnic minority groups and their subsequent inadequate and often harmful mental health care is a major factor driving the necessity to critique the way in which mental illness stigma narratives about ethnic minority populations have surfaced, developed and endured. Research studies from many countries including the USA (Williams et al., 2019), the UK (Bhui et al., 2018; Wallace et al., 2016; Weich et al., 2004) and New Zealand (Harris et al., 2006) show that ethnic minority people have higher levels of mental illness than their White counterparts, with racism shown to be a fundamental factor in worsening mental health (Paradies et al., 2015). Furthermore, ethnic minority people have worse experiences of mental health services. A global systematic review and meta‐analysis of ethnic inequalities in compulsory detention (Barnett et al., 2019) in psychiatric units showed that ethnic minority people (particularly people from Black and Asian backgrounds) were more likely to be compulsorily detained compared to their White counterparts (studies were included from the UK, Italy, Ireland, the Netherlands, USA, Switzerland, Denmark, Spain, Canada and New Zealand). In the UK specifically, research has shown ethnic inequalities in mental health service use to be widespread and largely unchanging. Many ethnic minority groups (compared to their White counterparts) are subject to increased diagnosis of schizophrenia (Halvorsrud et al., 2018); less likely to recover from an episode of mental illness and be less satisfied with services received (McManus et al., 2016); less likely to receive specialist treatment for mental health problems (Ahmad et al., 2021; Kapadia et al., 2018); and fearful of seeking help from mental health services due to past racist treatment from health‐care providers (Kapadia et al., 2022). The poorer mental health outcomes of ethnic minority people are therefore well‐established, but the way stigma has been postulated as a critical factor in driving these outcomes is somewhat misguided and potentially damaging.

In the UK, we see a growing proliferation of the idea that there is greater mental illness stigma in ethnic minority ‘communities’, referring largely to Black (both African and Caribbean) and South Asian (Pakistani, Indian and Bangladeshi) populations. The thesis tends to be that ‘culturally’ there is more stigma in these communities due to certain religious, spiritual or traditional beliefs about mental illness and the stigma that seeking help for these problems entails (Knifton, 2012; Shefer et al., 2013). These explanations are abundant not only among people who are suffering or who have suffered with mental illness, but also among their family members, health professionals, charities and advocacy groups working with marginalised ethnic minority groups. The recent UK Department of Health and Social Care White Paper, Reforming the Mental Health Act, placed stigma in ‘communities’ as one of the reasons for ethnic inequalities in mental health‐care pathways stating, ‘There are also cultural factors to consider, people from black, Asian and minority ethnic backgrounds may engage with services later, because of perceptions held within their communities for example, around recognising mental health problems early, on levels of associated stigma, as well as a distrust of services’ (Department of Health and Social Care, 2021). The same paper did not once refer to racism experienced by ethnic minority people. This is one example of how academic and public debates in this area have been skewed in favour of the idea that mental illness stigma is so great in ethnic minority populations that it can account for the inequalities we see in access to and experiences of mental health services with ethnic minority groups faring worse on many of these outcomes (Glover & Evison, 2009; Kapadia et al., 2022).

A review of the previous research shows that there is some evidence that mental illness stigma is greater in ethnic minority groups compared to White majority groups. The way stigma is defined in these studies varies greatly although the main types of stigma that they comment on can be broadly grouped into four categories [drawing on the work of Goffman (1963), Pescosolido & Martin (2015) and Corrigan et al., (2014)]: (1) public stigma (stereotypes, prejudice and discrimination enacted by the general public); (2) self‐stigma (internalised acceptance of stereotypes and prejudice); (3) courtesy stigma also known as affiliative stigma or stigma by association (Ostman & Kjellin, 2002) (stereotypes, prejudice and discrimination experienced by people associated with a person who has mental illness); and (4) structural stigma (stereotypes, prejudice and discrimination embedded into laws, policies, practices and enacted predominantly by public institutions). Three systematic reviews conducted in the last decade have stated that mental illness stigma is greater in ethnic minority groups. Two of these reviews (Clement et al., 2015; Eylem et al., 2020) include studies from around the world, whilst the other included US studies only (Misra et al., 2021). Eylem et al. (2020) meta‐analysed data from 29 studies reporting on public stigma and self‐stigma associated with common mental disorders and found that ethnic minority people displayed more stigma (public and self) than White majority people although the size of the difference was small (e.g. [overall effect size] = 0.20; 95% confidence interval [CI]: 0.12–0.27). Clement et al.'s (2015) review of 144 studies found that stigma (mainly self‐stigma) was associated with reduced help‐seeking for mental illness, and that ethnic minority people were disproportionately affected. However, the review did not include an effect size for White majority groups alongside ethnic minority groups for comparison purposes, so the magnitude of the disproportionality is unknown. Misra and colleagues’ review (2021) of US studies only aimed to identify ‘how cultural aspects of mental illness stigma manifest similarly and differently for racial and ethnic minority groups’ (p. 487). Ninety‐seven studies were included in the review and 25 of these were comparative (to the White American population); in these studies, ethnic minority groups (Asian Americans, Black Americans and Latin Americans) often expressed greater public or self‐stigma than White counterparts. Misra and colleagues’ review was the only one out of the three systematic reviews presented here that aimed to investigate all four types of stigma outlined above. Relatedly, many of the studies included in the reviews and other notable quantitative studies in the field that allow ethnic group comparisons (Anglin et al., 2006; Bhavsar et al., 2019; Conner et al., 2010; Corrigan & Watson, 2007; Loya et al., 2010; Nadeem et al., 2007) usually focus mainly on public stigma and self‐stigma and to a lesser extent on courtesy stigma.

There are also many qualitative studies in the field that purport to show that there are greater levels of stigma in ethnic minority populations (e.g., Alvidrez et al., 2008; Campbell & Mowbray, 2016; Knifton, 2012; Memon et al., 2016; Shefer et al., 2013; Tabassum et al., 2000; Vyas et al., 2021; Wood et al., 2022). These studies tend to draw conclusions both about the levels of stigma in certain ethnic minority populations and (over)state the importance of stigma in influencing mental health‐care pathways. For example, Campbell and Mowbray’s (2016) study of 17 Black Americans experiencing depression looked at the impact of stigma on help‐seeking and concluded that, ‘even though the stigma of mental illness is a barrier for many seeking services for mental health treatment, it is a particularly strong impediment for Black Americans’ (2016, p. 267). Similar conclusions are found in studies in the UK: Knifton’s (2012) study of stigma and its role in helpseeking amongst Chinese, Pakistani and Indian people living in Scotland states, ‘there was general agreement that mental health stigma and its consequences were profound and deeply embedded across and within the three communities’ (p. 292). However, almost none of these studies sample White populations (cf. Carpenter‐Song et al., 2010 for an exception) hence making it unreasonable to use these studies to suggest that levels of stigma are greater in ethnic minority groups. More importantly, these studies incorrectly make assertions about how mental illness stigma is a particularly problematic issue for ethnic minority groups and is posited as something that is more instrumental in mental health‐care pathways for these groups than for White majority groups. As can be seen from the studies detailed above, this serves to compound the racialisation (Nazroo, 1999) that ethnic minority people are already subjected to by implying that a person’s ethnic minority status and their related ‘culture’ and ‘cultural practices’ are the reasons for mental illness stigma in these minoritised groups (Kapadia & Bradby, 2021).

Measures of structural stigma are not often used in quantitative surveys, nor has it been considered in the qualitative studies cited above, indicating that this aspect of stigma is not considered to be as important as the others when theorising why ethnic minority people may not seek help from mental health services. This is a notable omission in the field which suggests that stigmatising actions resulting from stigma embedded in laws, policies and organisations do not shape mental health‐care pathways. Relatedly, it also suggests that other strongly related systemic processes such as structural and institutional racism in mental health services (evidenced by the inadequacy of previous, and the lack of current, race equality policies in mental health systems) are not key in explanations of lower use of mental health services by some ethnic minority groups. Although there have been calls in the field to (1) focus more on structural stigma (Hatzenbuehler, 2016; Tyler, 2020) and (2) consider how multiple aspects of a person’s social identity that are stigmatised (e.g., mental illness status and ethnic identity) can compound and worsen inequalities (Stangl et al., 2019; Turan et al., 2019), there is very little in the field that considers how structural aspects of mental health systems stigmatise ethnic minority people and use this ‘stigma power’ to ‘keep people down’ (Link & Phelan, 2014, p. 24). This is particularly important in relation to ethnic minority patients due to the racism they face both in the pursuit of seeking help for mental illness and in their subsequent mental health treatment.

## PROBLEMS WITH, AND PURPOSES OF, STIGMA NARRATIVES

The way in which the knowledge about mental illness stigma and ethnic minority groups has been used is worrying, as narratives have arisen suggesting that stigma is inherent in ethnic minority groups as well as suggestions that the solution to this ‘excessive stigma’ is targeted anti‐stigma interventions in these groups (Knifton, 2012; Shefer et al., 2013) in order to reduce ethnic inequalities in the use of mental health services. There has been very little consideration of the structural hierarchies and systems of power (i.e., racism) within which stigma operates. There are four main problems with prevailing stigma narratives as they relate to ethnic minority people, which are outlined below.

### Perpetuation of racialisation of ethnic minority groups

One of the fundamental questions that current mental illness stigma narratives raise is why is stigma postulated to be the main reason for low uptake of mental health services, but for ethnic minority groups only? The answer lies partially in the way in which ethnic minority people are racialised (see above). Within the mental health field, there is an erroneous tendency by some scholars and health professionals to unwittingly, or sometimes wittingly, locate the cause of mental illness within ethnic minority people themselves that is, people become ill *because* they are from a particular ethnic group, implying there is something genetically, biologically or culturally programed to cause ill health, which is specific to certain ethnic groups (Nazroo, 1998). This line of argument can also be seen in research on the use of mental health services, for example, Burr’s study  (2002) shows that some health professionals believed South Asian women in the UK did not access mental health services when needed due to their ‘culture’. Relatedly, there is a tranche of literature that asserts that mental illness may be both expressed in ‘culturally specific’ ways by some ethnic minority groups, for example, South Asian women are more likely to express mental illness via bodily symptoms (Ineichen, 1987; Krause, 1989) compared to White majority women. However, large‐scale national community studies that have compared the expression and measurement of mental illness between South Asian women and other women have shown that although there might be a slightly higher tendency for somatisation of symptoms for South Asian women (Das‐Munshi et al., 2014; Nazroo & O’Connor, 2002), this is not of the magnitude that has been suggested in other small, purposively sampled studies (Sheikh & Furnham, 2012; Wilson & MacCarthy, 1994). Further, in‐depth qualitative studies have shown that mental distress is expressed largely in the same way among South Asian women and White women with common terminology (Fenton & Sadiq‐Sangster, 1996; Mallinson & Popay, 2007).

Regardless of whether there are substantial differences in the expression of mental illness, their perceived aetiology and subsequent chosen treatment between ethnic minority and White majority groups, non‐Western forms of knowledge and understanding about mental illness are seen as inferior to Western forms of psychiatric knowledge (Fernando, 2017; Mills, 2014), and subsequently non‐Western ways of treating mental illness are seen as uncivilised, ineffective and ultimately based on incorrect understandings of the biological functioning of the human body. The consequences of branding alternative ways of thinking about mental illness as ‘faulty’ propagates the racialisation of ethnic minority people, by laying blame for the lack of help‐seeking (in Western Euro‐centric psychiatric systems such as those in the US and UK) on ethnic minority people. Further, ethnic minority people are stigmatised because they may hold these alternative views about mental illness and the supposed benefits of mental health services. However, the source of stigmatisation here is crucial; those working within the psychiatric system are the stigmatisers (Pilgrim & Rogers, 2005; Schulze, 2007), contrary to more popular narratives that posit ethnic minority people to be the source of mental illness stigma.

### The omission of racism

A notable omission in research in this field is consideration of (1) how racism impacts on ethnic minority people’s use of mental health services and (2) how mental illness stigma operates alongside racism. In relation to the first point, in particular, racial discrimination from health professionals has been downplayed as a major reason for under‐use of mental health services, despite robust evidence that racism exists within the structures of mental health services and is experienced in consultations with mental health professionals (Bhui et al., 2018; Fernando, 2017; Shim, 2021). A recent review of ethnic inequalities in mental health care in the UK showed that fear of racist treatment from health‐care staff was a major impediment to seeking help for mental health problems (Kapadia et al., 2022). The intense focus on stigma in ethnic minority groups (but not in White majority groups) as a reason for the lack of help‐seeking and poorer mental health outcomes is a deflection away from other problems that affect ethnic minority groups in the pursuit of improving their mental health. Using mental illness stigma as a deflection, in this way, is damaging to ethnic minority people’s health, as it focuses undue attention on reducing mental illness stigma within ethnic minority groups at the expense of ignoring other, more significant structural problems (mainly racism) that affect the poorer quality of mental health treatment that ethnic minority groups receive.

In relation to the second point about the lack of consideration of mental illness stigma alongside racism, the ‘double stigma’ (Gary, 2005, p. 979) faced as a consequence of having a mental illness and being from an ethnic minority background is not a new idea. It is, however, rather simple in its formulation and has not been sufficiently developed in order to give a full picture of what it means to be marginalised within two systems of oppression (stigma and racism). First, the argument considers only public, self‐stigma and courtesy stigma as influential in pathways to mental health care, thereby absolving health‐care professionals and the psychiatric system of their role in the (structural) stigmatisation of ethnic minority patients (this is addressed in more detail below). Second, the idea of double stigma implies that mental illness stigma and racism have equal effects on mental health outcomes, ignoring the arguably greater detrimental effect of racism on mental health treatment pathways and outcomes. Although, it is hard to quantify and compare the effects of stigma and racism within research studies, as large national survey datasets often do not measure both of these, and therefore analyses are not designed to ascertain the relative contribution of racism and stigma to mental health care. More broadly, this disregard of racism is also seen outside of academic research. In health policies and the agendas of large health and psychiatric professional bodies (World Health Organisation, 2021; World Psychiatric Association, 2019), mental illness stigma is a primary concern and therefore continues to feature heavily on research agendas and attract substantial amounts of funding. Racism, on the other hand, does not feature in global agendas in relation to mental health, although psychiatric bodies in some countries are now paying more attention to racism in mental health services, with the establishment of specialist committees to address racism in psychiatric services (e.g., American Psychiatric Association [Structural Racism Accountability Committee] in the US and the Royal College of Psychiatrists [Equality Action Plan] in the UK). But progress is slow and it seems unlikely that racism in mental health‐care pathways will be attended to in research and policy arenas (at some point in the near future) to the level that is required to meaningfully address ethnic inequalities. In order to ensure the research we produce is relevant, the way in which racism and structural stigma operate within mental health services must be brought together in order to understand how inequalities in access to mental health service and treatment pathways operate.

### Stigmatisation by professionals

As mentioned earlier in this article, structural stigma has received less attention in the field of mental illness stigma research, and correspondingly, there has been only marginal consideration of the stigmatisation enacted by psychiatrists and other mental health‐care professionals. There are varying reasons for the stigma that has been created by the mental health professionals, not least the desire for the psychiatric profession to elevate its standing within medicine and to give its methods and biomedical approach to treating ‘misery’ credence (Long, 2014). Furthermore, anti‐stigma campaigns have been argued to be ‘concealed attempt(s)’ (Schulze, 2007, p. 138) at raising the profile of psychiatry itself, rather than attempting to improve the mental health outcomes of patients (Pilgrim & Rogers, 2005). This is not altogether surprising since the deployment of stigma benefits those with power in social hierarchies with those at the top most likely to benefit; the ‘stigma power’ (Link & Phelan, 2014) yielded from mental illness stigma benefits the psychiatric profession much more than it benefits members of the lay public who may hold stigmatising views. Patients feel stigmatised by health professionals because of the way their mental illness is talked about (Mills, 2014; Pescosolido & Martin, 2015), but for ethnic minority patients this is compounded because of the way that they are also racialised in their treatments. One example of this is the racialisation of Black people evidenced by higher rates of diagnoses of psychotic illnesses such as schizophrenia for these groups (Halvorsrud et al., 2019; Schwartz, 2014). It can also be seen in the way Muslim patients are treated in the consulting room, where mental health‐care workers such as psychologists can become surveillants for the state (Younis & Jadhav, 2020) under anti‐terrorist programmes (such as Prevent in the UK). The Islamophobic basis underpinning such programmes mean that Muslim (and largely ethnic minority) patients are disproportionately negatively impacted in their treatment, in a way that non‐Muslim (mainly White) patients are not. The racist treatment of ethnic minority patients by the psychiatric profession also relates to the previous section in this article viz., how can we ensure both structural stigma and racism are addressed in the pursuit for racial equality in mental health services? Undoubtedly, the stigmatisation enacted by psychiatrists must be investigated more deeply in order to inform the conversations about stigma and ethnic minority people’s mental health but it would also be useful to consider ‘stigma as a technology of racism’ (Tyler, 2018, p. 747), one that operates within the psychiatric system is ‘deployed’ by mental health‐care professionals and renders those in lower positions of power to remain there (Tyler & Slater, 2018).

### Wrong solutions to the stigma problem

Given the lack of attention paid to the effects of structural stigma and racism on ethnic minority people’s mental health‐care pathways, it is perhaps not surprising that many of the solutions to the theorised stigma problem for ethnic minority people with mental illness are targeted at changing attitudes towards stigma in these populations or tailoring anti‐stigma campaigns (Conner et al., 2010; Eylem et al., 2020) specifically for ethnic minority groups. What is unclear is how they should be tailored and why this type of tailoring will be successful in reducing stigma in these groups? The reduction of public stigma in general population samples, although present, is small (Pescosolido et al., 2021; Walsh & Foster, 2021); and this is despite large financial resources dedicated to these anti‐stigma campaigns over many years. Hence, it seems that a different version of only marginally successful campaigns may not be the best strategy to improve ethnic inequalities in mental health care. Furthermore, as discussed in the previous section, the structural stigma experienced by ethnic minority people enacted by those in power (the psychiatric profession) is the source of some of the most detrimental outcomes for ethnic minority people, yet we are still seeing suggestions that individually targeted and tailored anti‐stigma interventions are the answer: an individual solution for a structural problem. This is akin to how individuals are targeted more generally by the psychiatric profession, which has worryingly close links with the pharmaceutical industry (Conrad, 2007), where people become the targets of medical interventions (particularly psychotropic medication) as a way of solving their problems that are caused by overarching inequitable institutional and structural systems (Tyler & Slater, 2018), yet the solution is packaged in pills, targeted at individuals’ biological systems.

## RECOMMENDATIONS TO REFOCUS RESEARCH ON STIGMA AND RACISM: CONTEXT, DATA AND ORIGINS

So how can we reframe, refocus and redirect theorisation and research on mental illness stigma in ethnic minority groups? It is important to point out that the aim of this article is not to deny the lived experience of mental illness of stigma for ethnic minority people, nor is it to deny that these experiences cause suffering for those people, their families and their friends. For us to reframe narratives in this field and begin to understand how they have flourished, for what purpose and why they continue to be so dominant in the field, a certain level of responsibility and sensitivity is required, that does not dismiss people who have experienced stigma. However, there needs to be a shift in the research field that positions the experiences of stigma for these groups in the context of other discrimination that they face, in order to move conversations away from individual reasons for the lack of help‐seeking to arguments about the structures and institutions that prohibit appropriate and efficacious mental health care for ethnic minority people. There are three main ways in which work in this field needs to be reshaped.

### Put stigma into a broader context

Research in the field should move towards looking at structural stigma and racism together to understand how both of these affect ethnic minority people’s pathways to mental health care and what kind of treatment they receive. The direction suggested here is what has been called for already in the field of mental illness stigma and stigma studies more generally (Hatzenbuehler, 2016; Turan et al., 2019), but we have yet to see a considerable shift. The ideas presented in this article provide a clear way forward to progress the field and refrain from using explanations based on stereotypes imbued with racism, as an explanation for ethnic differences in mental illness stigma. At present, studies continue to focus on public, self‐stigma or courtesy stigma, partly because of the tradition of using these measures in quantitative surveys and partly because there is a certain paradigm of using ‘cultural explanations’ by default, meaning that explanations do not move beyond looking at the individual and tend to pathologise certain ethnic minority groups. It is unsatisfactory to keep asking the same questions. And it is even more unsatisfactory to keep reproducing the same answers, that is,

Q1: Is there more stigma in ethnic minority groups? (Answer: Yes)
Q2: Why? (Answer: Due to cultural beliefs) and
Q3: What can we do about it? (Answer: We must tailor anti‐stigma campaigns to ethnic minority groups)

Certainly, ethnic differences in mental illness stigma should not be ignored but rather the conversations and research agenda around stigma need to be shifted considerably to bring the stigmatisation that is generated by mental health professionals and racism within the psychiatric system to the centre of the discussion. Efforts to reduce stigma also need to be shifted from focussing on individual patients to highlighting stigmatisation that is created and perpetuated by psychiatric systems.

### Design data collection appropriate for an examination of ethnic inequality

In order to robustly answer the types of questions based on the new areas of focus proposed in the preceding section, there needs to be well‐designed research samples. Quantitative surveys should collect large enough samples of ethnic minority groups (using ethnic minority boost sampling) that are of interest to the research study, as well as White majority comparative samples, in order to be able to make direct comparisons relating to the levels of mental illness stigma between ethnic groups. Too many national surveys do not contain large enough samples of ethnic minority respondents (Bécares et al., 2020), resulting in unreliable and imprecise estimates from statistical models using these data. Further, these surveys should ensure that measures of structural stigma (see Hatzenbuehler, 2017 for a thorough exposition of the ways in which measures of structural stigma could be operationalised) are collected as well as experiences of racism, both in interactions within and outside of the psychiatric and mental health‐care system, and preferably over the life course. Many surveys collect data on the most recent experiences of racism but evidence shows that cumulative experiences of racism have a dose‐response effect on mental illness (Wallace et al., 2016). Qualitative studies should also more fully consider recruiting White majority participants along with ethnic minority participants in order to delve deeper into ethnic differences in the way mental illness stigma is talked about and how it affects people. The questions that are asked of participants and the way that results are presented need to reflect a sharper focus on both structural stigma and racism, bringing both of these facets to the fore.

### Trace the origins of stigma narratives to understand their purpose and to begin to change them

In the pursuit to answer questions of how stigma affects the mental health‐care pathways of ethnic minority groups, a fundamental question relating to the origins of stigma narratives in ethnic minority populations has been relegated. In order to understand why stigma narratives persist, Tyler’s recent reconceptualisation of stigma is a major valuable addition to the field, and one that must be heeded particularly in relation to the subject matter of this article. For us to understand the entwined powerful roles of mental illness stigma and racism, we need to find out ‘where stigmatising attitudes come from, how and by whom is stigma crafted, mediated, produced and why, what social, political and economic functions stigmatisation might play in particular historical and geopolitical contexts, and how has stigma been resisted’ (Tyler & Slater, 2018, p. 736). Only then will we be able to begin to disrupt the existing damaging, dominant narratives about mental illness stigma in ethnic minority populations.

## CONCLUSION

Work that attempts to advocate for a shift of focus in well‐established sociological fields (such as mental illness stigma) may face a tough audience. However, in order to consider the role of stigma meaningfully in mental health‐care pathways for ethnic minority people, researchers in this field have a responsibility to ensure that they are asking the right research questions; by this, I mean that structural stigma and racism must be centralised in attempts to understand stigma narratives about ethnic minority populations. Additionally, the origins and purposes of these narratives must be thoroughly interrogated in order to understand how they have produced and continue to reproduce social and ethnic inequalities. Without this redirection and refocus in the field, research in the area runs the risk of further promulgating baseless ‘cultural explanations’ and simultaneously minimising the powerful role played by the psychiatric profession in the production and use of stigma narratives about ethnic minority people.

## AUTHOR CONTRIBUTIONS

**Dharmi Kapadia**: Conceptualization (Lead); Writing – original draft (Lead); Writing – review & editing (Lead).

## CONFLICT OF INTEREST STATEMENT

There are no conflicts of interest to declare.

## Data Availability

This is a review article; data have not been collected or analysed in the production of this paper.
